# Synergistic Effects of Fructose and Food Preservatives on Metabolic Dysfunction-Associated Steatotic Liver Disease (MASLD): From Gut Microbiome Alterations to Hepatic Gene Expression

**DOI:** 10.3390/nu16213722

**Published:** 2024-10-30

**Authors:** Tomas Hrncir, Eva Trckova, Lucia Hrncirova

**Affiliations:** Laboratory of Gnotobiology, Institute of Microbiology, Czech Academy of Sciences, 549 22 Novy Hradek, Czech Republic

**Keywords:** non-alcoholic fatty liver disease (NAFLD), fructose, food additives, gut microbiome, mycobiome, hepatic gene expression, intestinal permeability, inflammation

## Abstract

**Background/Objectives**: Metabolic dysfunction-associated steatotic liver disease (MASLD) is a growing global health problem closely linked to dietary habits, particularly high fructose consumption. This study investigates the combined effects of fructose and common food preservatives (sodium benzoate, sodium nitrite, and potassium sorbate) on the development and progression of MASLD. **Methods**: We utilized a human microbiota-associated mouse model, administering 10% fructose with or without preservatives for 11 weeks. Liver histology, hepatic gene expression (microarray analysis), biochemical markers, cytokine profiles, intestinal permeability, and gut microbiome composition (16S rRNA and Internal Transcribed Spacer (ITS) sequencing) were evaluated. **Results**: Fructose and potassium sorbate synergistically induced liver pathology characterized by increased steatosis, inflammation and fibrosis. These histological changes were associated with elevated liver function markers and altered lipid profiles. The treatments also induced significant changes in both the bacterial and fungal communities and disrupted intestinal barrier function, leading to increased pro-inflammatory responses in the mesenteric lymph nodes. Liver gene expression analysis revealed a wide range of transcriptional changes induced by fructose and modulated by the preservative. Key genes involved in lipid metabolism, oxidative stress, and inflammatory responses were affected. **Conclusions**: Our findings highlight the complex interactions between dietary components, gut microbiota, and host metabolism in the development of MASLD. The study identifies potential risks associated with the combined consumption of fructose and preservatives, particularly potassium sorbate. Our data reveal new mechanisms that are involved in the development of MASLD and open up a new avenue for the prevention and treatment of MASLD through dietary interventions and the modulation of the microbiome.

## 1. Introduction

Metabolic steatotic liver disease (MASLD) is a rapidly growing global health problem characterized by an excessive accumulation of fat in the liver in the absence of significant alcohol consumption. Recently, the term “metabolic dysfunction-associated steatotic liver disease” (MASLD) has been proposed to replace MASLD to better reflect the metabolic nature of the disease [1]. We will use this updated terminology throughout this manuscript. The global prevalence of MASLD is estimated to be 24% and rising, with the highest rates reported in the Middle East and South America [2]. MASLD encompasses a spectrum of conditions ranging from simple steatosis to non-alcoholic steatohepatitis (NASH), which can progress to fibrosis, cirrhosis, and hepatocellular carcinoma [3]. The disease is closely associated with symptoms of metabolic syndrome, including obesity, insulin resistance, and dyslipidemia [4].

The pathogenesis of MASLD is complex and multifactorial, involving interactions between genetic, environmental, and metabolic factors [5]. However, there is increasing evidence that the gut microbiota play a critical role in the development and progression of MASLD [6]. Alterations in gut microbial composition and function, known as dysbiosis, have been consistently observed in patients with MASLD [7]. These changes are characterized by reduced microbial diversity, shifts in the balance of beneficial and harmful bacteria, and altered microbial metabolic activities [8]. Recent research has highlighted the importance of environmental factors, particularly diet, in shaping the gut microbiome and influencing the pathogenesis of MASLD [9,10]. Of particular concern is the increased consumption of fructose and food additives in modern diets. Fructose, a major component of high-fructose corn syrup and sucrose, has been implicated in inducing gut dysbiosis, increasing intestinal permeability, and directly affecting liver metabolism [11]. Similarly, certain food additives have been shown to alter gut microbial communities and potentially exacerbate MASLD [12,13,14].

The gut–liver axis, a bidirectional communication system between the gastrointestinal tract and the liver, plays a critical role in the pathophysiology of MASLD [5]. The disruption of this axis, through increased intestinal permeability and the translocation of bacterial products, can lead to chronic low-grade inflammation and metabolic dysfunction in the liver [15]. Understanding the complex interplay between dietary factors, gut microbiota, and host metabolism is essential for the development of effective strategies for the prevention and treatment of MASLD [16].

This study aimed to investigate the combined effects of fructose and common food preservatives on the development and progression of MASLD. Our approach was to use a complex array of methods, including histological, biochemical, immunological, metataxonomic (16S rRNA and ITS sequencing), and genomic analyses, to elucidate the underlying mechanisms by which these dietary components affect gut microbiota composition, intestinal barrier function, and liver physiology. Our findings may provide new insights into the pathogenesis of MASLD and enable the development of novel preventive and therapeutic approaches targeting the diet–gut–liver axis.

## 2. Materials and Methods

### 2.1. Experimental Animals

Wild-type C57BL/6 mice were obtained from Jackson Laboratories and maintained under specific pathogen-free conditions. Germ-free C57BL/6 mice were generated and maintained in flexible film isolators in our gnotobiotic facility. Human gut microbiota-associated mice were generated by colonizing germ-free mice with a fecal sample obtained from a healthy human donor after appropriate informed consent and screening procedures. These mice were then bred in the facility for several generations to establish stable colonization. All mice were maintained on a 12 h light/dark cycle with ad libitum access to food and water. Both the mice in the breeding colonies and the experimental mice were fed a standard breeding diet (Cat. No. V1124, ssniff, Soest, Germany). For the experiments, all mice from the breeding colony that met the age criteria (3 weeks old) were included in the study. No animals were excluded from the analysis. The experimental mice were randomly assigned into five groups (water (control), fructose, fructose + benzoate, fructose + nitrite, and fructose + sorbate) and kept in airtight cages with high positive pressure and HEPA filters. Each group consisted of eight mice and the sexes were evenly divided. To minimize potential confounding factors, the order of treatments and measurements was randomized across groups. Humane endpoints were not established because the study procedures were not expected to cause significant pain or distress. Animals were monitored daily for general health and well-being. At the end of the study, mice were euthanized by cervical dislocation under isoflurane anesthesia. Animal care and experimental procedures were approved by the Institutional Animal Care and Use Committee and conducted in accordance with institutional guidelines.

### 2.2. Fructose and Preservative Supplementation

To induce MASLD, 10% fructose (*w*/*v*) was administered starting at 3 weeks of age for 11 weeks. The 11-week treatment period was chosen based on preliminary data showing sufficient development of MASLD symptoms in our mouse model. This time frame is consistent with the literature reporting MASLD development after 8–16 weeks of high-fructose diet feeding. Starting treatment at 3 weeks of age allowed us to cover the transition from adolescence to early adulthood (14 weeks of age at the end of experiment), a critical period for metabolic disease development. This duration balanced disease progression with efforts to minimize prolonged stress to animals. Fructose (Cat. No. F0127, Merck, Rahway, NJ, USA) was given ad libitum in the drinking water. The preservatives, namely sodium benzoate (E211; Cat. No. 71300, Merck), sodium nitrite (E250; Cat. No. 237213, Merck), and potassium sorbate (E202; Cat. No. 85520, Merck), were administered together with the fructose. Exposure to the preservatives, normalized to mouse body weight and water consumption, was adjusted to the estimated maximum daily intake of the additives in the European population (source: Report from the Commission on Dietary Food Additive Intake in the European Union, https://publications.europa.eu, accessed on 12 March 2023). These were 4.8 mg/kg bw/day for benzoate, 0.36 for nitrite, and 19.0 for sorbate. All solutions were freshly prepared each week and kept refrigerated until use.

### 2.3. Histological Sample Preparation and Staining

Liver tissue samples were fixed in 10% neutral buffered formalin, dehydrated through a graded ethanol series, cleared in xylene substitute (Neo-Clear, Merck), and embedded in paraffin according to standard protocols. Sections were cut at 5 μm thickness and mounted on glass slides. H&E staining was performed according to standard procedures. Briefly, sections were deparaffinized, rehydrated, stained with Mayer’s hematoxylin, counterstained with eosin, dehydrated, cleared, and mounted. Masson’s trichrome staining was performed according to the manufacturer’s protocol (Diapath S.p.A., Martinengo, Italy). The deparaffinized and rehydrated sections were stained with Weigert’s iron hematoxylin for 10 min, followed by picric acid solution for 4 min. The sections were then stained with Biebrich’s scarlet acid fuchsin for 4 min and differentiated in phosphomolybdic acid solution for 10 min. Finally, the sections were counterstained with aniline blue for 4 min, dehydrated through a graded series of ethanol, cleared, and mounted. This procedure stains nuclei black, muscle fibers and cytoplasm red, and collagen fibers blue. All histological slides were evaluated by an experienced histopathologist in a blinded manner.

### 2.4. Biochemical Analyses

The plasma levels of liver enzymes and lipids were measured using commercially available kits from Merck (Rahway, NJ, USA). Alanine aminotransferase (ALT) activity was measured using the ALT Activity Assay Kit (Cat. No. MAK052, Merck, Rahway, NJ, USA). Aspartate aminotransferase (AST) activity was measured using the AST Activity Assay Kit (Cat. No. MAK055, Merck, Rahway, NJ, USA). Alkaline phosphatase (ALP) activity was measured using the ALP Activity Assay Kit (Cat. No. MAK447, Merck, Rahway, NJ, USA). Triglyceride levels were quantified using the Triglyceride Quantification Assay Kit (Cat. No. MAK266). Total and free cholesterol levels were determined using the Cholesterol Quantitation Kit (Cat. No. MAK043, Merck, Rahway, NJ, USA). All assays were performed according to the manufacturer’s instructions. Absorbance measurements were recorded using a SPECTROstar Nano microplate reader (BMG LABTECH, Ortenberg, Germany). Standard curves were generated for each assay to calculate the concentrations of the respective analytes in the plasma samples.

### 2.5. Oral Administration of FITC–Dextran and Measurement of Plasma Fluorescence to Determine Intestinal Permeability

Intestinal permeability was determined in vivo using FITC-labeled dextran (4 kDa, Merck). Mice were fasted for 4 h before the oral administration of 200 μL FITC–dextran solution (50 mg/mL in PBS) per 20 g body weight. After 4 h, mice were briefly anesthetized with isoflurane to minimize pain and distress. Blood was then collected from the submandibular vein and allowed to clot for 30 min at room temperature. Plasma was separated by centrifugation at 2000× *g* for 10 min at 4 °C. Plasma samples were diluted with PBS (35 μL plasma in 175 μL PBS) and fluorescence was measured using a Qubit fluorometer (Thermo Fisher Scientific, Waltham, MA, USA) with blue excitation at 470 nm and green emissions at 510 and 580 nm. A standard curve was generated via the serial dilution of FITC–dextran in a mixture of plasma and PBS (17% plasma in PBS) to determine the final concentration of FITC–dextran in the plasma samples. Higher concentrations of FITC–dextran in plasma indicate increased intestinal permeability.

### 2.6. Sequencing of the 16S rRNA Gene and the Internal Transcribed Spacer (ITS) Amplicon and Bioinformatic Analysis

The composition of the microbial community was analyzed by sequencing the 16S rRNA gene and the ITS amplicon. DNA was extracted using the QIAamp PowerFecal DNA Kit (QIAGEN, Hilden, Germany). Amplicon libraries targeting the V3-V4 region of the 16S rRNA gene (341f—806bR primers) and the ITS1 region (ITS1F—ITS2 primers) were prepared. The libraries were sequenced on the Illumina MiSeq platform (2 × 300 bp) using MiSeq reagent V3 (Illumina, San Diego, CA, USA).

Bioinformatic analysis was performed using QIIME 2 version 2024.5 [17]. Raw reads were demultiplexed and the raw read quality was visualized using FastQC version 0.12.1 [18]. Primer sequences were trimmed using cutadapt version 4.5 [19]. Reads were denoised, chimeras were removed, and amplicon sequence variants (ASVs) were identified using the DADA2 version 1.28 workflow [20]. For 16S data, a multiple sequence alignment was performed using MAFFT version 7.520 [21] and a phylogenetic tree was constructed using FastTree 2.1.11 [22].

Taxonomy was assigned to ASVs using a naive Bayes classifier trained on the SILVA database release 138 for 16S [23] or the UNITE database version 9.0 for ITS. Unassigned ASVs and contaminants of mitochondrial and chloroplast origin were removed. Alpha diversity metrics, including Shannon’s diversity index, observed ASVs, Faith’s phylogenetic diversity (for 16S only), and Pielou’s evenness, were calculated after rarefaction. Beta diversity metrics, including Jaccard distance, Bray–Curtis dissimilarity, unweighted UniFrac, and weighted UniFrac (for 16S only), were calculated to compare community composition between sample groups. Differential abundance analysis was performed using ANCOM-BC2 version 1.2.0 [24] with a significance threshold of W-statistic > 0.7 and FDR-adjusted *p*-value < 0.05. Taxonomic composition was visualized using interactive stacked bar plots. Additional statistical analyses and data visualization were performed using R version 4.1.0 [25] in RStudio version 2024.04.2 [26].

### 2.7. Cell Isolation and Culture

Harvested tissues (spleen, mesenteric lymph nodes, and liver) were placed in separate tissue culture dishes containing cold PBS. Each tissue was gently disrupted into a single cell suspension using the plunger of a 2 mL syringe. The resulting suspensions were filtered through 70 μm cell strainers (BD Biosciences, Franklin Lakes, NJ, USA) and washed twice with PBS via centrifugation at 400× *g* for 5 min at 4 °C. Red blood cells were lysed with ACK lysis buffer (150 mM NH_4_Cl, 10 mM KHCO_3_, 0.1 mM EDTA, pH 7.2) for 5 min at room temperature, followed by two washes with PBS. Liver cell suspensions were further purified by gradient centrifugation on 33% Percoll (GE Healthcare) at 500× *g* for 20 min at room temperature without break. The interface containing lymphocytes was collected and washed twice with PBS. Isolated cells were resuspended in complete RPMI-1640 medium supplemented with 10% heat-inactivated fetal bovine serum (FBS), 2 mM L-glutamine, 100 U/mL penicillin, and 100 μg/mL streptomycin. Cells were cultured at a density of 1 × 10^6^ cells/mL in 24-well plates and stimulated with phorbol 12-myristate 13-acetate (PMA, 20 ng/mL) and ionomycin (500 ng/mL) for 5 h at 37 °C in a humidified incubator with 5% CO_2_.

### 2.8. Cytokine Profiling by ELISA

Cytokine levels were measured in cell supernatants using ThermoFisher ELISA kits (Thermo Fisher Scientific, Waltham, MA, USA) according to the manufacturer’s protocols. The cytokines quantified included IFNγ, TNFα, IL-6, IL-10, and IL-17A. Briefly, 96-well plates were coated overnight at 4 °C with 100 μL/well of capture antibody diluted in coating buffer. After three washes with wash buffer (PBS + 0.05% Tween-20), the plates were blocked with 200 μL/well assay diluent for 1 h at room temperature. Standards and samples were diluted in assay diluent as needed and 100 μL was added to the appropriate wells. The plates were incubated for 2 h at room temperature. After three washes, 100 μL of detection antibody diluted in assay diluent was added to each well and incubated for 1 h. After three washes, 100 μL of diluted streptavidin–HRP was added to each well and incubated for 30 min. After five washes, 100 μL of TMB substrate solution was added to each well and the plates were allowed to develop for 10–30 min. The reaction was stopped by the addition of 100 μL stop solution (1 M H_2_SO_4_). Absorbance was measured at 450 nm with wavelength correction at 620 nm. Cytokine concentrations were calculated from four-parameter logistic standard curves. Samples were run in duplicate. All samples below the lowest standard were assigned the value of the minimum detectable concentration for that assay.

### 2.9. RNA Extraction and Quality Control

Total RNA was extracted from mouse liver tissue samples stabilized in RNAlater buffer. Extraction was performed using the RNeasy Micro Kit (QIAGEN, Hilden, Germany) according to the manufacturer’s protocol for “Purification of total RNA from animal and human tissue”. Briefly, tissue samples were disrupted and homogenized in 600 μL RLT buffer containing 1% β-mercaptoethanol using a Precellys 24 Homogenizer (Bertin Corp., Rockville, MD, USA) with Precellys CK14 ceramic beads (2 cycles of 15 s at 6500 rpm with a 30 s break). After centrifugation, 300 μL of the clarified lysate was processed through RNeasy MinElute spin columns (QIAGEN, Hilden, Germany), including an on-column DNase digestion step. Total RNA was eluted in 14 μL nuclease-free water. RNA integrity and purity were assessed using an Agilent 2100 Bioanalyzer with the RNA 6000 Nano LabChip reagent set (Agilent, Palo Alto, CA, USA).

### 2.10. Gene Expression Profiling by Microarray

Gene expression profiling was performed using Applied Biosystems GeneChip Clariom S mouse arrays. Sample preparation and microarray hybridization were performed according to the manufacturer’s protocol (GeneChip WT PLUS Reagent Kit, Thermo Fisher Scientific, Waltham, MA, USA). Briefly, 200 ng of total RNA was used to generate double-stranded cDNA. After purification, 20 μg of cRNA was synthesized by in vitro transcription and then used to generate single-stranded (ss) cDNA incorporating dUTP. The purified ss cDNA was fragmented using uracil DNA glycosylase and apurinic/apyrimidinic endonuclease 1 and terminally labeled with biotin. A total of 3.8 μg of fragmented and labeled ss cDNA was hybridized to the arrays for 16 h at 45 °C with rotation at 60 rpm. Arrays were washed and stained using an Applied Biosystems GeneChip Fluidics Station 450 and scanned using an Applied Biosystems GeneChip Scanner 3000 7 G system. Fluidics and scanning functions were controlled by the Applied Biosystems GeneChip Command Console v5.0 software.

### 2.11. Microarray Data Analysis

Microarray data analysis was performed using the Transcriptome Analysis Console (TAC) 4.0.2 software (Applied Biosystems). Probe set signal values were calculated using the Signal Space Transformation-Robust Multi-Chip Analysis (SST-RMA) algorithm. Differential gene expression analysis was performed using the Linear Models for Microarray Data (LIMMA) method. Genes were considered differentially expressed if they met the following criteria: fold change < −2 or >2 and false discovery rate (FDR) *p*-value < 0.05. Principal component analysis and hierarchical clustering were used to visualize sample relationships.

### 2.12. Statistical Analysis

Data are presented as mean ± standard deviation (SD) unless otherwise noted. Statistical analyses were performed using GraphPad Prism 10 for macOS (GraphPad Software, San Diego, CA, USA), QIIME2 software package (version 2024.5, www.qiime2.org (accessed on 13 June 2024)), Transcriptome Analysis Console (TAC) 4.0.2 software (Thermo Fisher Scientific, Waltham, MA, USA), and R studio (version 2024.04.2, Boston, MA, USA). One-way analysis of variance (ANOVA), followed by Tukey’s post hoc test for multiple comparisons, was used for comparisons between groups. For the microbiome data analysis, alpha diversity metrics were compared using the Kruskal–Wallis test, while beta diversity was assessed using PERMANOVA with 999 permutations. A differential abundance analysis of bacterial taxa was performed using ANCOM-BC2 with a significance threshold of W-statistic > 0.7 and FDR-adjusted *p*-value < 0.05. For gene expression data, differential expression analysis was performed using the limma-voom pipeline in R, with significance thresholds of |log2 fold change| > 1 and an FDR-adjusted *p*-value < 0.05. Significance levels are reported as follows: * *p* ≤ 0.05, ** *p* ≤ 0.01, *** *p* ≤ 0.001, and **** *p* ≤ 0.0001. To account for multiple comparisons in our analyses, we used appropriate correction methods across our various datasets. For microbiome data analysis, we used the Benjamini–Hochberg procedure to control for the false discovery rate (FDR) when comparing alpha diversity metrics and in the ANCOM-BC2 differential abundance analysis. For gene expression data, we used the Benjamini–Hochberg method to adjust *p*-values in our differential expression analysis. In our cytokine profiling experiments, we used the Bonferroni correction when performing multiple t-tests. Adjusted *p*-values (q-values) are reported throughout the Section 3, and only results that were significant after correction are discussed as primary results. Sample size (*n* = 8 per group) was determined based on power analysis using G*Power software version 3.1.9.6. Parameters were set to detect a standardized effect size of 1.5 at 80% power and α = 0.05 for primary outcomes. This sample size is consistent with similar studies and balances statistical power with ethical considerations to minimize animal use.

## 3. Results

### 3.1. Fructose and Preservatives Synergistically Induce Hepatic Steatosis and Inflammation

**Figure 1:**
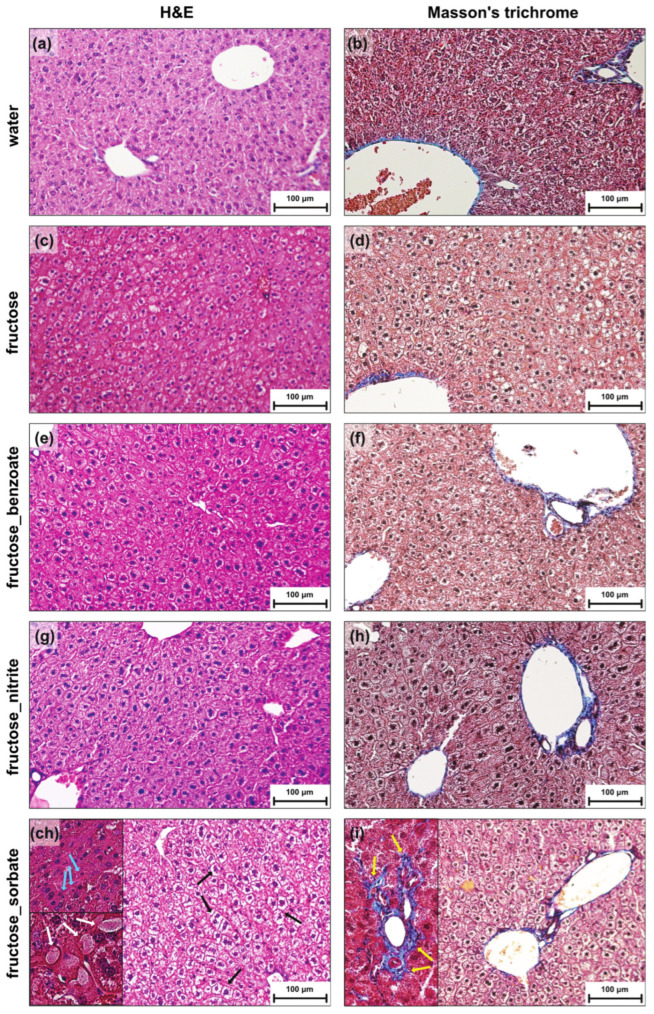
Histological analysis of liver sections from mice treated with fructose in combination with preservatives. Representative H&E and Masson’s trichrome stained liver sections from (**a**,**b**) control mice and mice treated with (**c**,**d**) fructose, (**e**,**f**) fructose + benzoate, (**g**,**h**) fructose + nitrite, and (**ch**,**i**) fructose + sorbate. Scale bars represent 100 μm. (**a**,**b**) Water-treated control liver shows normal hepatic architecture with no evidence of steatosis, inflammation, or fibrosis. (**c**–**i**) Livers from mice treated with fructose alone and in combination with preservatives exhibit varying degrees of steatosis, characterized by the presence of lipid droplets within the hepatocytes. (**ch**) Livers from mice treated with fructose + sorbate show an extensive steatosis with ballooning degeneration of hepatocytes (black arrows), disruption of normal lobular architecture, an infiltration of mononuclear inflammatory cells (upper inset, blue arrows), glycogen deposition (lower inset, white arrows), and (**i**) early stages of fibrosis (left inset, yellow arrows).

A histological analysis of liver sections revealed significant differences between the treatment groups (Figure 1). Water-treated control mice showed normal liver architecture without steatosis, inflammation, or fibrosis (Figure 1a,b). In contrast, mice treated with fructose alone or in combination with preservatives showed varying degrees of hepatic steatosis characterized by lipid droplets within hepatocytes (Figure 1c–i). The combination of fructose and sorbate resulted in the most severe liver pathology (Figure 1ch,i). These liver sections showed significant infiltration with mononuclear inflammatory cells, disrupted lobular architecture (Figure 1ch, upper inset), and hepatocyte ballooning degeneration (Figure 1ch, main image). Glycogen deposition was also observed, indicating altered hepatic metabolism (Figure 1ch, bottom inset). Notably, early-stage fibrosis was evident in this group (Figure 1i), suggesting potential progression to advanced liver disease.

These findings demonstrate that while fructose alone can induce hepatic steatosis, its combination with preservatives, particularly sorbate, exacerbates liver damage. The synergistic effects that are observed highlight the importance of considering the combined effects of dietary components and food additives on liver health.

### 3.2. Fructose Consumption Alters Liver Function and Lipid Metabolism, and Food Preservatives May Modulate These Effects

**Figure 2:**
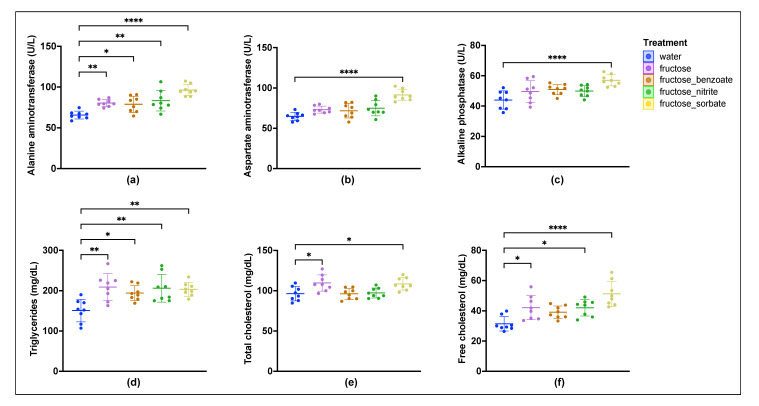
Effects of fructose and food preservatives on liver function markers and lipid profiles in mice. Plasma levels of liver enzymes and lipids were measured. (**a**) Alanine aminotransferase (ALT), (**b**) aspartate aminotransferase (AST), (**c**) alkaline phosphatase (ALP), (**d**) triglycerides, (**e**) total cholesterol, and (**f**) free cholesterol. Data are expressed as mean ± SD. Statistical analysis was performed by one-way ANOVA followed by Tukey’s multiple comparison test. Asterisks indicate significant differences compared with the water control group: * *p* ≤ 0.05, ** *p* ≤ 0.01, and **** *p* ≤ 0.0001.

Fructose administration resulted in significant increases in plasma ALT, triglycerides, and cholesterol levels compared to the water control (Figure 2). The addition of food preservatives to fructose resulted in different effects. Notably, only the addition of sorbate resulted in increases in all three liver enzymes, i.e., ALT, AST, and ALP, whereas the addition of benzoate and nitrite significantly increased only ALT. Triglyceride levels were increased in all treated groups. Total cholesterol levels were moderately elevated in the fructose and fructose–sorbate groups. Free cholesterol levels were significantly elevated in the fructose and fructose–nitrite groups and highly elevated in the fructose–sorbate group. These results suggest that the addition of food preservatives, especially sorbate, leads to an amplification of the negative effects of fructose on liver function and lipid metabolism.

### 3.3. Fructose and Food Additives Synergistically Increase Intestinal Permeability

**Figure 3:**
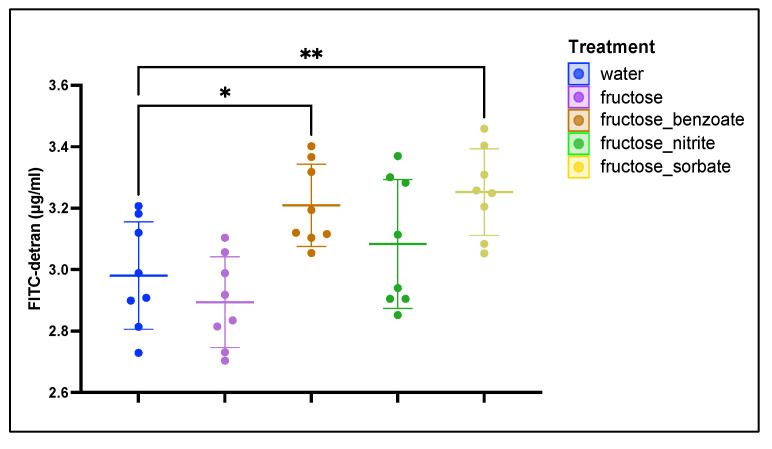
Intestinal permeability measured by FITC–dextran absorption. Intestinal permeability was determined by the oral administration of FITC–dextran (4 kDa) and subsequent measurement of plasma fluorescence after 4 h. Mice were treated with water (control), fructose alone, or fructose in combination with benzoate, nitrite, or sorbate. Data are presented as mean plasma FITC–dextran concentrations (μg/mL) ± standard deviation. Statistical significance was determined by one-way ANOVA followed by Dunnett’s post hoc test. * *p* < 0.05, ** *p* < 0.01. *n* = eight mice per group.

Intestinal permeability was determined by measuring plasma FITC–dextran concentration after oral administration (Figure 3). Fructose treatment did not significantly increase intestinal permeability compared to the water control group (2.89 ± 0.15 μg/mL vs. 2.98 ± 0.18 μg/mL). However, when fructose was combined with food additives, permeability increased. The most pronounced increase in permeability was observed in the fructose plus sorbate group (3.25 ± 0.15 μg/mL, *p* < 0.01 vs. water control), followed by fructose plus benzoate (3.21 ± 0.14 μg/mL, *p* < 0.05). These results suggest that the combined consumption of fructose and preservatives increases intestinal permeability.

### 3.4. Fructose Combined with Sorbate Alters Fungal but Not Bacterial Alpha Diversity

**Figure 4:**
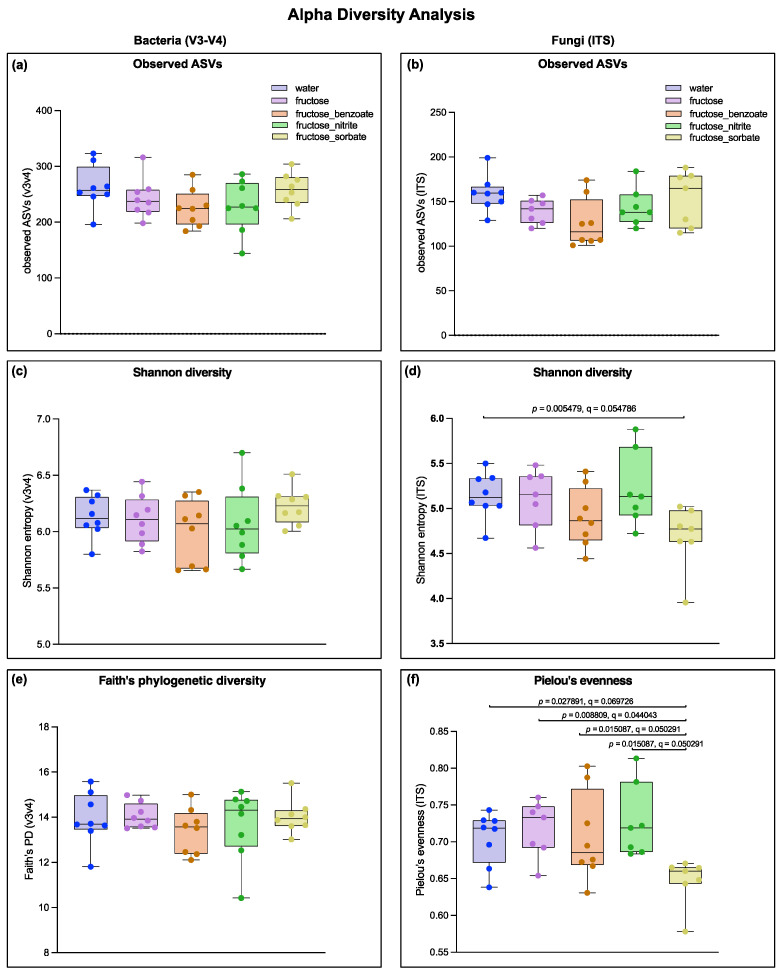
Analysis of alpha diversity of bacterial (16S V3-V4) and fungal (ITS) communities. Measures of bacterial alpha diversity: (**a**) observed ASVs, (**c**) Shannon diversity index, and (**e**) Faith’s phylogenetic diversity. Measures of alpha diversity for fungi: (**b**) observed ASVs, (**d**) Shannon diversity index, and (**f**) Pielou’s evenness. Shannon diversity index represents both the richness and evenness of species, while Faith’s Phylogenetic diversity considers the phylogenetic differences between species. Higher values indicate greater diversity. Pielou’s evenness ranges from 0 to 1, with 1 indicating complete evenness in species abundance. Each point represents a single sample, with boxplots showing the median and interquartile range. The colors indicate different treatment groups: water (control), fructose, fructose + benzoate, fructose + nitrite, and fructose + sorbate. *n* = eight mice per group. Statistically significant differences (*p* < 0.05) between groups are indicated by *p*-values and q-values (FDR-corrected *p*-values). Bacterial alpha diversity metrics showed no significant differences between treatment groups. In contrast, fungal communities showed significant differences in Shannon diversity (*p* = 0.005479, q = 0.054768) and Pielou’s evenness (*p* = 0.027891, q = 0.069726) for the fructose–sorbate group compared to the control group, suggesting that the treatments had a stronger effect on fungal community structure than on bacterial community structure. Data were analyzed using QIIME2 and graphs were generated using GraphPad Prism 10.

The analysis of alpha diversity metrics revealed different responses between bacterial and fungal communities to the treatments. Bacterial communities showed surprising resilience, with no significant differences in observed ASVs, Shannon diversity index, or Faith’s phylogenetic diversity across any treatment groups (Figure 4a,c,e). In contrast, fungal communities showed significant sensitivity, but only to the fructose–sorbate treatment. This group showed significantly lower Shannon diversity (*p* = 0.005479, q = 0.054786) and Pielou’s evenness (*p* = 0.027891, q = 0.069726) compared to the water control and other treatment groups (Figure 4d,f). Notably, fructose alone or in combination with benzoate or nitrite did not significantly alter fungal alpha diversity metrics. These results suggest that while bacterial community richness and diversity remained stable across all treatments, the combination of fructose and sorbate uniquely disrupted the fungal community.

### 3.5. Fructose and Food Preservatives Induce Significant Shifts in the Composition of the Bacterial and Fungal Community

**Figure 5:**
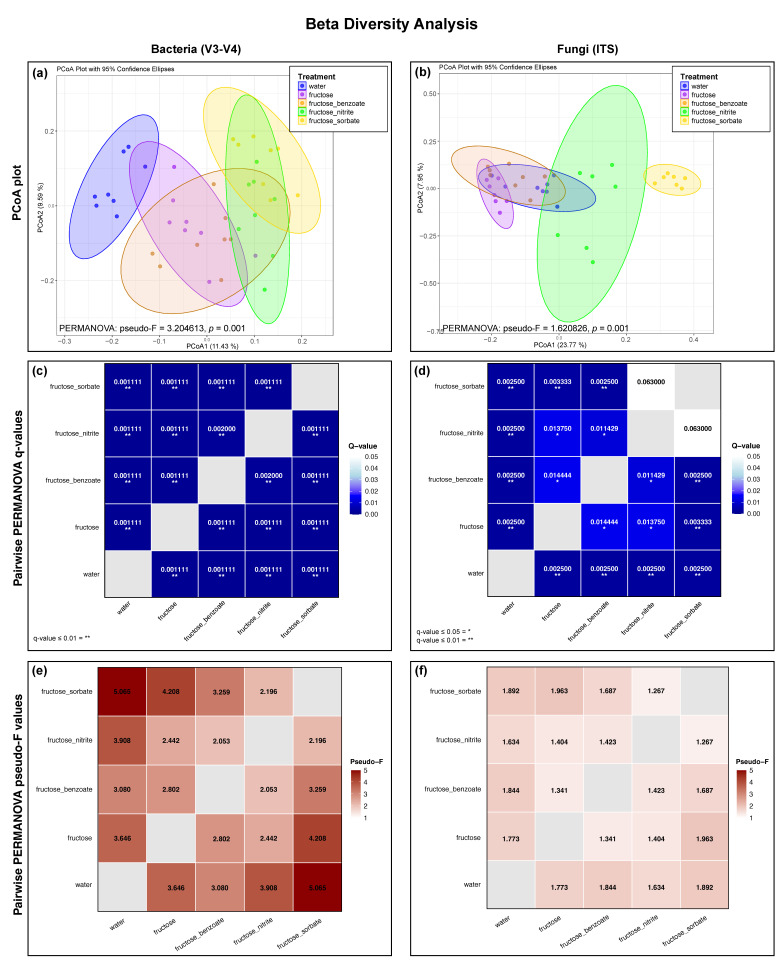
Beta diversity analysis of bacterial (16S V3-V4) and fungal (ITS) communities. (**a**,**b**) Principal Coordinate Analysis (PCoA) plots based on weighted UniFrac distances for bacteria and Bray–Curtis dissimilarities for fungi. Each point represents one sample, with colors indicating treatment groups. The closer the points, the more similar the community composition. (**c**–**f**) Heatmaps showing pairwise PERMANOVA results. Darker colors in (**c**,**d**) indicate lower q-values (stronger statistical significance), while darker colors in (**e**,**f**) indicate higher pseudo-F values (larger effect sizes). PERMANOVA analysis shows the significant effects of treatments on both bacterial and fungal communities. Pairwise comparisons also revealed significant differences (q < 0.05) between most treatment groups for both bacterial and fungal communities. Greater differentiation was observed for bacterial communities (lower q values and higher pseudo-F values). Data were analyzed using QIIME2 and plots were generated using R Studio.

Beta diversity analysis revealed significant differences in the composition of bacterial and fungal communities between treatment groups. For bacterial communities, Principal Coordinate Analysis (PCoA) based on weighted UniFrac distances showed a clear separation between treatment groups (Figure 5a), which was confirmed by PERMANOVA (pseudo-F = 3.204613, *p* = 0.001). Pairwise comparisons revealed significant differences (q < 0.05) between all treatment groups, with a particularly strong differentiation observed between the water control and the fructose–sorbate treatment (Figure 5c,e).

Fungal communities also showed significant shifts in composition between treatments, as shown by PCoA based on Bray–Curtis dissimilarities (Figure 5b) and confirmed by PERMANOVA (pseudo-F = 1.620826, *p* = 0.001). Pairwise comparisons showed significant differences between most treatment groups (Figure 5d,f), although the strength of differentiation was generally lower than for the bacterial communities, as indicated by the higher q values and lower pseudo-F values.

### 3.6. Fructose in Combination with Benzoate or Sorbate Induces the Greatest Changes in Bacterial Taxa Abundance

**Figure 6:**
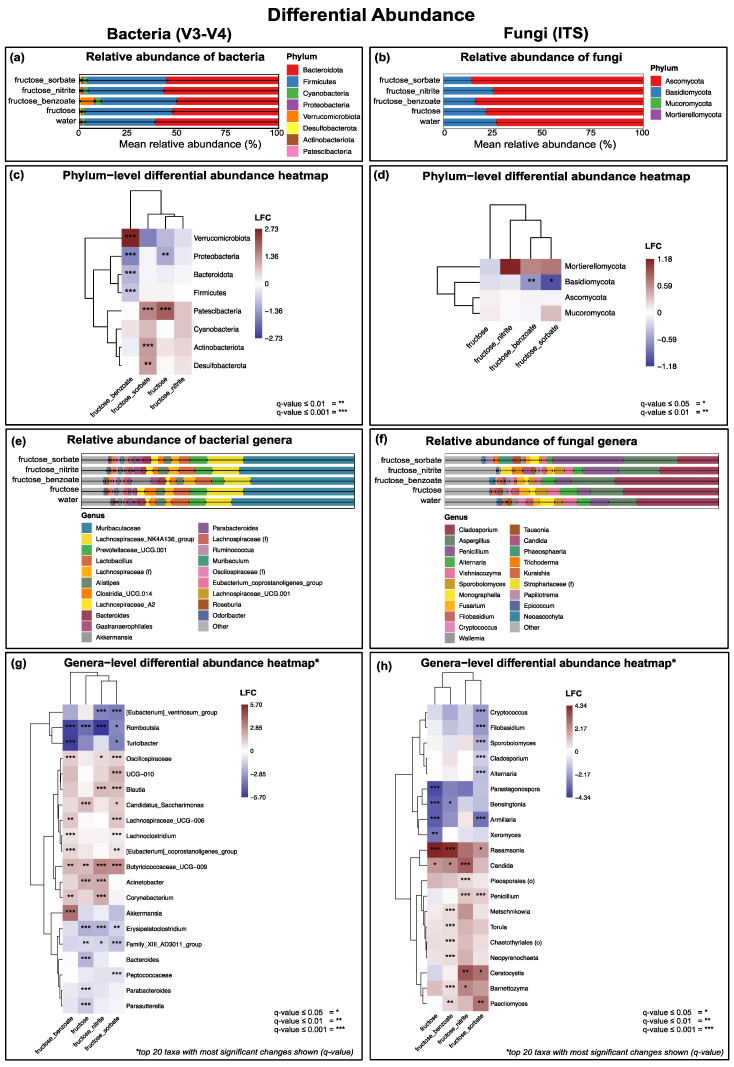
Differential abundance analysis of bacterial (16S V3-V4) and fungal (ITS) communities. (**a**,**c**,**e**,**g**) Bacterial community analysis: (**a**) stacked bar graph showing the relative abundance of bacterial phyla across treatment groups; (**c**) heatmap showing differential abundance at the phylum level, where colors represent log fold changes (LFC) relative to the water control group; (**e**) stacked bar graph showing the relative abundance of the top 20 bacterial genera across treatment groups; (**g**) heatmap showing differential abundance at the genus level for the top 20 taxa with the most significant changes (based on q-values), where colors represent log fold changes (LFC) relative to the water control group. (**b**,**d**,**f**,**h**) Fungal community analysis: (**b**) stacked bar plot showing relative abundance of fungal phyla across treatment groups; (**d**) heatmap showing differential abundance at the phylum level, where colors represent log fold changes (LFC) relative to the water control group; (**f**) stacked bar graph showing the relative abundance of the top 20 fungal genera across treatment groups; (**h**) heatmap showing differential abundance at the genus level for the top 20 taxa with the most significant changes (based on q-values), where colors represent log fold changes (LFC) relative to the water control group. For both bacterial and fungal heatmaps, statistical significance is indicated by asterisks: * q ≤ 0.05, ** q ≤ 0.01, *** q ≤ 0.001. Treatment groups are indicated as water (control), fructose, fructose + benzoate, fructose + nitrite, and fructose + sorbate. *n* = eight mice per group. Bacterial communities showed significant changes in all phyla except Cyanobacteria, with notable changes at the genus level in *Akkermansia*, *Acinetobacter*, Butyricicoccaceae, *Blautia*, and many others. Fungal communities showed significant shifts, mainly in the Basidiomycota phyla, with genus-level changes observed in *Rasamsonia*, *Candida*, and *Penicillium*, among others. The data were analyzed using QIIME2 and graphs were generated using R studio.

Differential abundance analysis revealed that the effects on bacterial taxa varied considerably between treatments. At the phylum level, fructose alone induced significant changes in two phyla (an increase in Patescibacteria and a decrease in Proteobacteria), demonstrating its ability to modulate the gut microbiome. Interestingly, fructose combined with nitrite showed no significant effect on any phylum, suggesting a potential neutralizing effect of nitrite on fructose-induced changes. The most profound changes were observed in the fructose–benzoate and fructose–sorbate groups. The fructose–benzoate combination significantly affected four phyla, causing an increase in Verrucomicrobia and a decrease in Proteobacteria, Bacteroidota (formerly Bacteroidetes), and Firmicutes. The fructose–sorbate group induced changes in three phyla, with increases in Patescibacteria, Actinobacteria, and Desulfobacteria. A complex pattern of changes was observed at the genus level. Significant increases were detected in several genera, including *Akkermansia* (especially in the fructose-benzoate group), *Corynebacterium*, *Acinetobacter*, Butyricicoccaceae, *Lachnoclostridium*, Lachnospiraceae, *Blautia*, and Oscillospiraceae. Conversely, significant decreases were observed in genera such as *Romboutsia* and *Turicibacter* (Figure 6g).

These results highlight that while fructose alone can induce some changes in bacterial abundance, its combination with certain food additives, particularly benzoate and sorbate, leads to more extensive and diverse changes in fecal bacterial community structure. This suggests a synergistic effect between fructose and these additives in modulating the gut microbiome, which could have important implications for host health and metabolism.

### 3.7. Fructose and Food Additives Selectively Alter the Abundance of Fungal Taxa

An analysis of the fungal communities revealed selective and treatment-specific changes in the abundance of taxa. At the phylum level, significant changes were observed mainly in the Basidiomycota (Figure 6d). Notably, both the fructose–benzoate and fructose–sorbate treatments induced a decrease in this phylum, suggesting a consistent effect of these combinations on fungal community structure. At the genus level, we observed a complex pattern of changes that varied by treatment. Fructose alone caused the most significant depletion of fungal genera, particularly *Parastagonospora*, *Bensingtonia*, *Armillaria*, and *Xeromyces*, while increasing the abundance of *Rasamsonia*. The fructose–benzoate combination also induced an increase in *Rasamsonia*. In contrast, the fructose–nitrite increased *Candida*, *Penicillium*, and *Ceratocystis*. The fructose–sorbate treatment shared some effects with fructose–nitrite, causing increases in *Penicillium* and *Ceratocystis*. However, it also caused the most extensive depletion of fungal genera, including *Cryptococcus*, *Filobasidium*, *Sporobolomyces*, *Cladosporium*, *Alternaria*, and *Armillaria*. These distinct patterns of changes in fungal taxa highlight the specific and diverse effects of fructose, both alone and in combination with different food preservatives. The observed shifts in both bacterial and fungal communities suggest that these treatments, particularly the combinations of fructose with benzoate and sorbate, significantly affect the composition of the gut microbiome. These changes could potentially influence host–microbe interactions and metabolic processes, highlighting the importance of considering both bacterial and fungal components when studying the effects of dietary factors on the gut microbiome.

### 3.8. Fructose and Food Preservatives Induce Organ-Specific Changes in Cytokine Profiles

**Figure 7:**
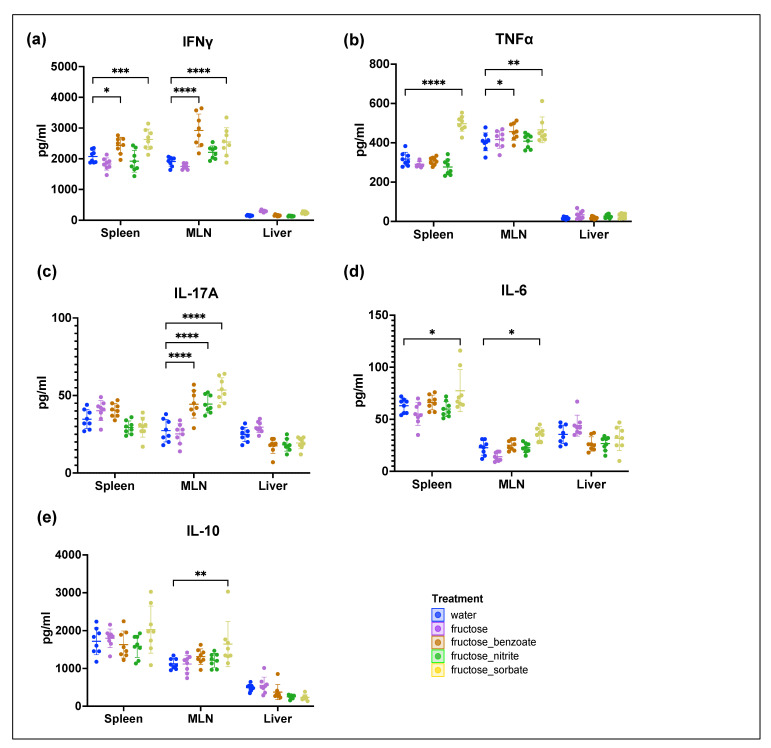
Cytokine profiles of multiple organs in response to fructose and preservative treatments. Cytokine levels were measured in the spleen, mesenteric lymph nodes (MLN), and liver tissues using ELISA. The mice were treated with water (control), fructose alone, or fructose in combination with preservatives (benzoate, nitrite, or sorbate). Quantified cytokines include (**a**) IFNγ, (**b**) TNFα, (**c**) IL-17A, (**d**) IL-6, and (**e**) IL-10. Data are presented as mean ± SD in pg/mL. Statistical significance was determined using two-way ANOVA followed by Tukey’s post hoc test. * *p* < 0.05, ** *p* < 0.01, *** *p* < 0.001, **** *p* < 0.0001. *n* = eight mice per group.

To investigate the effects of fructose and food preservatives on immune responses, we measured cytokine levels in the spleen, mesenteric lymph nodes (MLN), and liver of treated mice using ELISA (Figure 7). Our results showed different patterns of cytokine production in the different organs and treatments. Fructose alone had no significant effect on any of the cytokines measured. The most pronounced effect was observed with the fructose–sorbate treatment, which stimulated the production of IFNγ, TNFα, and IL-6 in both spleen and MLN. In the MLN, this treatment also stimulated the production of IL-10 and IL-17A. The fructose–benzoate treatment stimulated the production of IFNγ in the spleen and of IFNγ, TNFα, and IL-17A in the MLN. The fructose–nitrite treatment significantly increased cytokine production only in the MLN. Collectively, these results show that fructose–preservative combinations, especially fructose–sorbate, induce a pro-inflammatory cytokine profile in multiple organs. This profile is characterized by increased levels of IFNγ, TNFα, IL-6, and IL-17A. The increase in IL-10 production could play a counter-regulatory role and potentially attenuate the pro-inflammatory response. The organ-specific nature of these changes, particularly the pronounced effects in the MLN, suggests that gut-associated lymphoid tissue plays a critical role in mediating immune responses to fructose and food additives. This finding highlights the potential importance of the gut–immune axis in the physiological response to food components [10,27]. The lack of detectable cytokine changes in the liver may be due to methodological limitations. The higher dilutions required for liver samples due to the lower numbers of isolated liver leukocytes may have resulted in cytokine levels falling below the detection limit of the ELISA method. These results provide important insights into the immunomodulatory effects of fructose–preservative combinations and highlight the need for further investigation of their potential impact on systemic and organ-specific immune responses.

### 3.9. Fructose Induces Significant Changes in Hepatic Gene Expression

**Figure 8:**
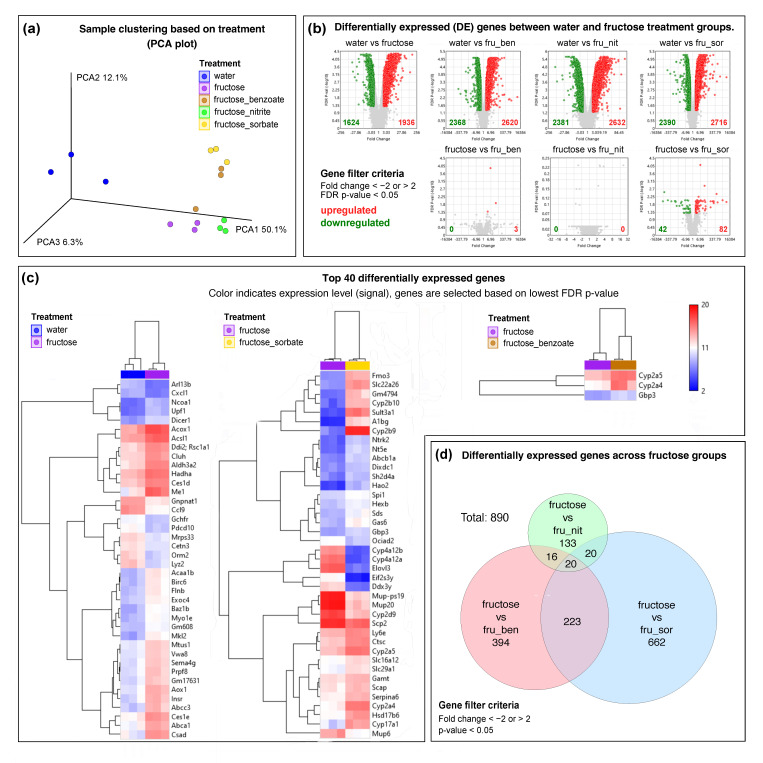
Gene expression analysis in liver tissue across different fructose-based treatments. (**a**) PCA plot showing sample clustering based on treatment groups (three mice/group). Each dot represents a sample, and colors indicate different treatments. The percentage of variance explained by each principal component is shown on the axes. (**b**) Volcano plot showing the differentially expressed genes between the water and fructose treatment groups. The *x*-axis represents the fold change in expression, while the *y*-axis shows the −log10 of the FDR *p*-value. Each dot represents a gene, with red dots indicating significantly up-regulated genes and green points indicating significantly down-regulated genes. The genes were filtered based on the following criteria: fold change < −2 or >2; FDR *p*-value < 0.05. (**c**) Hierarchical clustering heatmap of top 40 differentially expressed genes between water- and fructose-based treatments. The colors indicate the expression level (signal), with genes selected based on the lowest FDR *p*-value. Clustering was performed on both genes and samples using Euclidean distance metric and the complete linkage method (maximum distance between pairs of objects in clusters). The length of the dendrogram branches represents the degree of similarity between clusters, with shorter branches indicating more closely related objects. (**d**) Venn diagram showing the overlap of differentially expressed genes across all fructose–preservative treatment comparisons. Numbers indicate unique and shared differentially expressed genes between groups. Gene filter criteria: Fold change < −2 or >2; FDR *p*-value < 0.05.

To investigate the effects of fructose consumption on hepatic gene expression, we performed RNA sequencing on liver samples from mice treated with water (control) or fructose. Principal component analysis (PCA) of the gene expression data revealed a clear separation between the control and fructose-treated groups along the first principal component (PCA1), which accounted for 50.1% of the variance (Figure 8a). This indicates that fructose treatment caused a significant shift in the overall hepatic transcriptome.

Differential expression analysis identified 3 560 genes that were significantly altered by fructose treatment compared to the water control (fold change < −2 or >2, FDR *p*-value < 0.05) (Figure 8b). Of these, 1936 genes were upregulated and 1624 were downregulated. The top differentially expressed genes are shown in the heatmap (Figure 8c), which highlights the different expression patterns between the water and fructose treatments.

### 3.10. Key Genes Associated with the Pathogenesis of MASLD Were Significantly Modulated by Fructose

Significant changes were found in the expression of several genes known to be associated with the development of MASLD (Supplementary Table S1). The upregulation of Me1 (22-fold) and Acsl1 (4.2-fold) indicates increased lipogenesis and fatty acid activation, respectively. Me1 provides NADPH for de novo lipogenesis, while Acsl1 activates long-chain fatty acids and facilitates their incorporation into triglycerides. These changes likely contribute to the observed hepatic steatosis that is a hallmark of MASLD. The 20-fold upregulation of the insulin receptor gene (Insr) may indicate the development of insulin resistance, a key feature of MASLD pathogenesis.

Regarding oxidative stress, the 17-fold upregulation of Aox1, which is involved in ROS production, indicates increased oxidative stress, which can lead to lipid peroxidation and cellular damage. The concurrent 6.2-fold increase in Aldh3a2, which protects against lipid peroxidation, may represent a compensatory mechanism, although it may not be sufficient to fully counteract the increased oxidative stress.

Interestingly, some changes appeared to be potentially protective. The 25-fold increase in Csad, which is involved in the biosynthesis of taurine, may be beneficial, as taurine deficiency is associated with MASLD. Similarly, the 11-fold increase in Abca1, which is involved in cholesterol efflux, could help reduce cholesterol accumulation. The unexpected decrease in the pro-inflammatory chemokines Cxcl1 and Ccl9 (by 4.4-fold and 10-fold, respectively) requires further investigation as it may represent an early compensatory response or a more complex modulation of the inflammatory process in the development of MASLD.

### 3.11. Food Preservatives Modulate Fructose-Induced Gene Expression Changes

PCA revealed that although all fructose-treated groups clustered separately from the water control, there were significant differences between the fructose-only and the fructose–preservative groups (Figure 8a). This suggests that the preservatives had additional effects on gene expression beyond those induced by fructose alone. Differential expression analysis showed that each preservative uniquely modulated the fructose-induced gene expression profile (Figure 8c). Compared to fructose alone, sodium benzoate altered the expression of 394 genes, sodium nitrite altered the expression of 133 genes, and potassium sorbate altered the expression of 662 genes (fold change < −2 or >2, *p*-value < 0.05) (Figure 4d).

Of particular interest, several cytochrome P450 enzymes showed dramatic changes in expression upon preservative treatment (Supplementary Table S1). For example, Cyp4a12b and Cyp14a12a were strongly downregulated in the fructose–sorbate group compared to fructose alone (by −717-fold and −2499-fold, respectively), whereas Cyp2b9 was strongly upregulated (by 6356-fold). These enzymes play a crucial role in xenobiotic and lipid metabolism, suggesting that preservatives may significantly affect these processes in the context of fructose-induced metabolic changes.

Other notable genes affected by preservatives included Elovl3 (involved in fatty acid elongation), which was downregulated by 1835-fold, and Sult3a1 (involved in lipid metabolism), which was upregulated by 2267-fold in the fructose plus sorbate group. These changes highlight the potential of food preservatives to affect lipid metabolism in the fructose-exposed liver.

In conclusion, our results show that fructose consumption induces widespread changes in hepatic gene expression that are consistent with the development of MASLD. Furthermore, we show for the first time that common food preservatives can significantly modulate these fructose-induced changes, which may alter the course of MASLD progression. These findings highlight the need for further investigation of the combined effects of fructose and food preservatives on liver health.

## 4. Discussion

This study provides new insights into the synergistic effects of fructose and food preservatives on the pathogenesis of MASLD. While fructose alone induced liver injury, its combination with certain preservatives, particularly potassium sorbate, resulted in more pronounced liver injury and triggered extensive changes in the gut microbiota and host immune responses. Histological analysis revealed that the combination of fructose and sorbate caused the most severe liver pathology, with significant steatosis, inflammatory cell infiltrates, and early-stage fibrosis. This synergistic effect was also supported by biochemical analyses, which showed that the fructose–sorbate combination led to the most pronounced increases in liver enzymes (ALT, AST, ALP) and changes in lipid profiles. These findings are consistent with previous studies demonstrating the hepatotoxic potential of fructose [11] and highlight the potential of common food preservatives to amplify these effects.

Metataxonomic analysis revealed differential effects on bacterial and fungal communities. Interestingly, bacterial alpha diversity remained stable across all treatments, but fungal diversity was significantly reduced by the fructose–sorbate combination. This highlights the need to study both the bacterial and fungal components of the gut microbiome. It should be emphasized that the study of the fungal microbiome, an often-overlooked factor, should be an integral part of MASLD research [28]. The observed reduction in fungal diversity could have important implications for metabolic health, given the emerging role of the mycobiome in regulating host metabolism [29].

Beta-diversity analyses revealed significant shifts in the composition of both bacterial and fungal communities. The fructose–sorbate and fructose–benzoate combinations caused the most extensive changes, especially in the bacterial phyla Verrucomicrobia, Proteobacteria, and Bacteroidota. The increase in Verrucomicrobia was remarkable and was mainly caused by the expansion of the genus *Akkermansia*. The increase in Akkermansia was the most prominent in the fructose–benzoate group. While *Akkermansia muciniphila* is often considered beneficial in metabolic disorders due to its ability to improve intestinal barrier function and reduce inflammation [30], its excessive increase (over four-fold in our study) may have paradoxical effects. As a mucolytic bacterium, the *Akkermansia* overgrowth could potentially compromise the mucus layer, leading to increased intestinal permeability, which is consistent with our findings on intestinal permeability. This could facilitate the translocation of bacterial products and contribute to liver inflammation and the progression of MASLD. The observed changes in specific microbial taxa are likely to have a significant functional impact on host metabolism and the progression of MASLD.

Of particular concern is the increased abundance of *Candida* observed with all fructose-based treatments. Recent studies have shown that MASLD patients have an altered mycobiome, leading to increased immune responses to *Candida albicans* [31,32]. This alteration may contribute to systemic inflammation and metabolic dysregulation. *Candida* species are known to affect the host metabolism through multiple mechanisms, including alterations in glucose homeostasis and the lipid metabolism, which may exacerbate MASLD. In addition, the reduced fungal diversity observed in the fructose sorbate group may indicate a loss of beneficial fungal species that play a role in maintaining metabolic health. This reduction in diversity could lead to a less resilient gut ecosystem, potentially making the host more susceptible to metabolic disorders and inflammatory stimuli.

Taken together, these microbial shifts suggest a dysbiosis that favors a pro-inflammatory, metabolically dysregulated gut environment. The altered microbial community likely affects the host metabolism through multiple pathways, including alterations in short-chain fatty acid production, bile acid metabolism, and intestinal barrier integrity. Future studies should focus on investigating the specific metabolites of these altered microbial communities and their direct effects on liver function and lipid metabolism.

Our study revealed an interesting pattern in intestinal permeability. While fructose alone did not significantly increase permeability, its combination with preservatives, particularly sorbate and benzoate, led to a significant increase. This suggests that preservatives enhance the ability of fructose to disrupt the intestinal barrier function, possibly facilitating the translocation of bacterial products and contributing to liver inflammation [33].

Cytokine profiling experiments provided valuable insights into the immunomodulatory effects of fructose–preservative combinations. The organ-specific nature of these effects, with the mesenteric lymph nodes (MLN) showing the most pronounced changes, suggests a critical role for gut-associated lymphoid tissue in mediating immune responses to these dietary components. The fructose–sorbate combination induced a pro-inflammatory cytokine profile characterized by increased levels of IFNγ, TNFα, IL-6, and IL-17A, which may contribute to the exacerbation of liver injury [34].

Gene expression analysis revealed extensive transcriptional changes induced by fructose and further modulated by preservatives. The upregulation of genes involved in lipid metabolism (e.g., Me1, Acsl1) and oxidative stress (e.g., Aox1) is consistent with the known mechanisms of fructose-induced liver injury [35]. However, the observed upregulation of potentially protective genes (e.g., Csad, Abca1) suggests the activation of compensatory mechanisms that should be further investigated. Importantly, our study demonstrates for the first time that common food preservatives can significantly modulate fructose-induced changes in hepatic gene expression. The dramatic changes in the expression of cytochrome P450 enzymes after treatment with preservatives, particularly sorbate, suggest that these additives may profoundly affect xenobiotic and lipid metabolism in the context of fructose-induced metabolic changes.

The results of the gene expression analysis show that fructose induces extensive changes in gene transcription that are further modulated by preservatives. The overexpression of genes associated with the fatty acid metabolism (e.g., ME1 and ACSL1) and reactive oxygen species (ROS) production (e.g., AOX1) is consistent with the established mechanisms of fructose-induced liver injury [31]. However, the observed increase in the expression of potentially protective genes, including Csad and Abca1, suggests that compensatory mechanisms may be activated, which warrants further investigation. Notably, this study demonstrates for the first time that common food preservatives can significantly modulate fructose-induced changes in hepatic gene expression. The significant shifts in the expression of cytochrome P450 enzymes following the administration of preservatives, particularly sorbate, suggest that these additives may have a profound effect on xenobiotic and lipid metabolisms.

Our findings are consistent with and extend recent research on the role of diet and the gut microbiome in MASLD. A comprehensive review by Semmler et al. [36] highlighted the importance of dietary interventions in the treatment of MASLD, with a focus on calorie restriction and adherence to a Mediterranean diet. Our study provides experimental evidence to support these dietary recommendations by demonstrating the detrimental effects of high fructose consumption, particularly when combined with food preservatives. While Semmler et al. discussed the potential benefits of intermittent fasting in reducing liver fat content, our findings suggest that certain food additives may counteract these benefits by disrupting metabolic homeostasis and altering the gut microbiome. The review also highlighted the emerging role of personalized nutrition based on genetic background and microbiome composition. Our findings on the differential effects of preservatives on gut bacterial and fungal communities highlight the complexity of diet–microbiome interactions and underscore the need for personalized approaches to the treatment of MASLD. By elucidating the synergistic effects of fructose and preservatives on hepatic gene expression and the gut microbiota, our study provides new insights into the mechanisms by which modern dietary patterns may contribute to the progression of MASLD and builds on the current understanding of the role of diet in liver health.

Our data suggest several potential mechanisms by which fructose and preservatives may synergistically promote the progression of MASLD: (a) increased hepatic lipid accumulation due to the upregulation of lipid metabolism genes; (b) mitochondrial dysfunction and cellular damage due to increased oxidative stress; (c) intestinal barrier dysfunction leading to the increased translocation of bacterial products and liver inflammation; (d) altered xenobiotic and lipid metabolism due to changes in the expression of cytochrome P450 enzymes. The development and progression of MASLD are likely facilitated by these interrelated pathways. Future research should focus on confirming these mechanistic links by examining how these pathways are inhibited or activated.

Our human microbiota-associated mouse model provides valuable insights into the interactions between diet, gut microbiota, and host metabolism in the context of MASLD. However, we acknowledge several limitations that may complicate the direct translation of our findings into human physiology. Although, the doses of fructose (10% *w*/*v* in drinking water) and food preservatives used in our study were chosen to model high fructose consumption and the maximum allowable intake of preservatives in humans, they may not perfectly reflect the complexity and variability of the human diet. In addition, human dietary habits and metabolism may differ significantly from those of mice. In addition, our study design focused on the effects of preservatives in a fructose-induced MASLD model without pure preservative groups, which limited our ability to study isolated preservative effects. We also acknowledge that our histological analysis was performed only on paraffin-embedded, H&E-, and Masson trichrome-stained specimens and not on frozen sections, which would have provided additional confirmation of hepatic steatosis. Finally, although our sample size was sufficient to detect significant effects for the primary outcomes, future studies with larger animal cohorts may reveal additional subtle effects of the fructose–preservative combinations.

Despite these limitations, our findings on the synergistic effects of fructose and preservatives on the gut microbiota, intestinal permeability, and hepatic gene expression provide important mechanistic insights that are likely to be relevant to the pathogenesis of MASLD. Several human studies have shown an association between high fructose intake and the development of MASLD [11,35,37]. There is also increasing evidence that food additives may affect the human gut microbiota and its physiology [12,13,38].

Future research should also include preservative-only groups to gain a full understanding of the independent and synergistic effects of these additives on the development of MASLD. It would be useful to conduct longitudinal studies to investigate the long-term effects of the observed changes in gut microbiota, immune function, and hepatic gene expression, and to validate the findings in clinical trials. In addition, future research should focus on the investigation of the underlying molecular mechanisms.

Our findings have important implications for public health and dietary recommendations. The identified synergistic effects of fructose with specific preservatives highlight the urgent need to re-evaluate existing dietary guidelines. It is imperative that regulatory agencies evaluate the individual effects of food additives and their potential interactions with other dietary components in order to establish safe consumption levels for these dietary components.

These findings underscore the importance of encouraging the consumption of whole, minimally processed foods, not only in patients with MASLD, but also in the general public. We also believe that healthcare providers should advise MASLD patients to minimize their consumption of food preservatives, especially when consuming foods that are high in fructose.

The results of our study highlight the possibility of using microbiome modulation as a therapeutic approach for the prevention and treatment of MASLD. Targeted probiotic interventions, such as supplementation with specific *Lactobacillus* or *Bifidobacterium* strains, may prove to be an effective strategy to restore beneficial bacterial populations and reduce pro-inflammatory effects. Prebiotic supplements have the potential to stimulate the growth of beneficial microbes, which may improve gut barrier function and reduce inflammation. In addition to prebiotic supplementation, a diet that is rich in complex carbohydrates and fermentable fiber may naturally promote the development of a more diverse and resilient gut microbiome. Given the observed increase in oxidative stress, antioxidant therapies may prove beneficial in reducing liver damage. These potential intervention strategies need to be further investigated in both preclinical and clinical studies to determine their efficacy in preventing and treating MASLD associated with high fructose and preservative consumption.

## 5. Conclusions

Our findings reveal the existence of synergistic effects of dietary fructose and food preservatives, particularly potassium sorbate, and their involvement in the pathogenesis of MASLD. We demonstrated that these dietary components collectively induce potentially deleterious changes in the gut microbiota, disrupt intestinal barrier function, and induce liver damage. These findings suggest that dietary guidelines for the management of MASLD should consider not only macronutrient composition, but also the presence of common food additives.

We believe that our study provides valuable mechanistic insights. However, we must acknowledge several limitations. The use of a human microbiota-associated mouse model, while sophisticated, may not fully represent human MASLD pathogenesis. In addition, the fructose and preservative doses used may not perfectly reflect human dietary patterns. Our study design, which did not include preservative-only groups, limits our ability to differentiate the independent effects of these additives from their synergistic interactions with fructose.

These findings suggest several directions for future research. Clinical trials are needed to validate our findings in human populations and to explore potential therapeutic interventions targeting the identified pathways. Long-term studies that examine the effects of the combined consumption of fructose and preservatives would provide valuable insights into the consequences of chronic exposure. Future experimental designs should include preservative-only groups and use metagenomic and metabolomic approaches to better understand the functional implications of the observed microbial changes.

## Data Availability

The original contributions presented in the study are available in the article/Supplementary Materials. The raw metagenomics and gene expression data have been deposited in the NCBI repositories and are publicly available. The 16S rRNA and ITS sequencing data have been deposited in the NCBI Sequence Read Archive (SRA) under BioProject accession number PRJNA1148934, accessible at https://trace.ncbi.nlm.nih.gov/Traces/study/?acc=PRJNA1148934&o=acc_s%3Aa (accessed on 8 August 2024). Gene expression data from the Clariom S arrays are available in the NCBI Gene Expression Omnibus (GEO) under accession number GSE275135, which can be accessed at https://www.ncbi.nlm.nih.gov/geo/query/acc.cgi?acc=GSE275135 (accessed on 8 August 2024). These datasets are available for use by researchers in accordance with the terms of the respective repositories.

## Supplementary Materials

The following supporting information can be downloaded at: https://www.mdpi.com/article/10.3390/nu16213722/s1, Table S1: Key gene expression changes in fructose-induced MASLD model. The modulating effect of food preservatives.
